# Racial and Ethnic Differences in Mobile App Use for Meeting Sexual Partners Among Young Men Who Have Sex With Men and Young Transgender Women: Cross-Sectional Study

**DOI:** 10.2196/54215

**Published:** 2024-09-11

**Authors:** Kathryn Risher, Patrick Janulis, Elizabeth McConnell, Darnell Motley, Pedro Alonso Serrano, Joel D Jackson, Alonzo Brown, Meghan Williams, Daniel Mendez, Gregory Phillips II, Joshua Melville, Michelle Birkett

**Affiliations:** 1 Department of Public Health Sciences, Penn State University College of Medicine, Hershey, PA, United States; 2 Department of Medical Social Sciences, Northwestern University Feinberg School of Medicine, Chicago, IL, United States; 3 Department of Psychology, Palo Alto University, Palo Alto, CA, United States; 4 Department of Medicine, University of Chicago, Chicago, IL, United States; 5 Department of Preventive Medicine, Northwestern University Feinberg School of Medicine, Chicago, IL, United States; 6 Office of Diversity, Equity and Inclusion, Urban Health Initiative, University of Chicago Medicine, Chicago, IL, United States; 7 GoodCompany, Chicago, IL, United States; 8 Chicago House and Social Service Agency, Chicago, IL, United States; 9 EDDR Corporation, Chicago, IL, United States

**Keywords:** young men who have sex with men and young transgender women, hookup or dating apps, sexual partners, race and ethnicity, race, ethnicity, mobile app, racial bias, sexual partner, young, transgender, Chicago, United States, online, dating app, racism, analysis, youth, social network, hookup, black, Hispanic, Tinder, Grindr, Scruff, sexual mixing patterns, sexual patterns, sexual pattern, sexually transmitted infection, sexually transmitted infections

## Abstract

**Background:**

Young men who have sex with men and young transgender women (YMSM-YTW) use online spaces to meet sexual partners with increasing regularity, and research shows that experiences of racism online mimics the real world.

**Objective:**

We analyzed differences by race and ethnicity in web-based and mobile apps used to meet sexual partners as reported by Chicago-based YMSM-YTW in 2016‐2017.

**Methods:**

A racially and ethnically diverse sample of 643 YMSM-YTW aged 16‐29 years were asked to name websites or mobile apps used to seek a sexual partner in the prior 6 months, as well as provide information about sexual partnerships from the same period. We used logistic regression to assess the adjusted association of race and ethnicity with (1) use of any website or mobile apps to find a sexual partner, (2) use of a “social network” to find a sexual partner compared to websites or mobile apps predominantly used for dating or hookups, (3) use of specific websites or mobile apps, and (4) reporting successfully meeting a sexual partner online among website or mobile app users.

**Results:**

While most YMSM-YTW (454/643, 70.6%) used websites or mobile apps to find sexual partners, we found that Black non-Hispanic YMSM-YTW were significantly less likely to report doing so (comparing White non-Hispanic to Black non-Hispanic: adjusted odds ratio [aOR] 1.74, 95% CI 1.10‐2.76). Black non-Hispanic YMSM-YTW were more likely to have used a social network site to find a sexual partner (comparing White non-Hispanic to Black non-Hispanic: aOR 0.20, 95% CI 0.11‐0.37), though this was only reported by one-third (149/454, 32.8%) of all app-using participants. Individual apps used varied by race and ethnicity, with Grindr, Tinder, and Scruff being more common among White non-Hispanic YMSM-YTW (93/123, 75.6%; 72/123, 58.5%; and 30/123, 24.4%, respectively) than among Black non-Hispanic YMSM-YTW (65/178, 36.5%; 25/178, 14%; and 4/178, 2.2%, respectively) and Jack’d and Facebook being more common among Black non-Hispanic YMSM-YTW (105/178, 59% and 64/178, 36%, respectively) than among White non-Hispanic YMSM-YTW (6/123, 4.9% and 8/123, 6.5%, respectively). Finally, we found that while half (230/454, 50.7%) of YMSM-YTW app users reported successfully meeting a new sexual partner on an app, Black non-Hispanic YMSM-YTW app users were less likely to have done so than White non-Hispanic app users (comparing White non-Hispanic to Black non-Hispanic: aOR 2.46, 95% CI 1.50‐4.05).

**Conclusions:**

We found that Black non-Hispanic YMSM-YTW engaged with websites or mobile apps and found sexual partners systematically differently than White non-Hispanic YMSM-YTW. Our findings give a deeper understanding of how racial and ethnic sexual mixing patterns arise and have implications for the spread of sexually transmitted infections among Chicago’s YMSM-YTW.

## Introduction

Young men who have sex with men and young transgender women (YMSM-YTW) primarily use websites and mobile apps to find sexual partners [1]. However, there is a body of evidence that suggests that racial and ethnic minority YMSM-YTW experience online dating differently than majority populations [2-5]. In particular, sexual minority men of color report online exclusion through sexual racism [2], decreased sexual capital [5], and fetishization [3], as well as additional domains of rejection and degradation [4]. Further, transgender women of color seeking relationships may experience both dehumanizing stereotypes and sexual objectification, which may be tied to gender-based violence [6]. People belonging to multiple marginalized identities experience intersectional stigma in their use of mobile dating apps [3]. These differential experiences likely lead racial and ethnic minority YMSM-YTW to interact with mobile dating apps differently than White non-Hispanic YMSM-YTW, but there is insufficient evidence to support this claim.

Sexual racism is common among men who have sex with men (MSM) and transgender women of color of all ages both online and offline. A premobile dating app study in San Francisco found Black MSM specifically were reported as less “preferred” partners [7]. Similarly, in a study of MSM recruited on a mobile app in 2015, respondents reported a dis-preference for Black and Asian men as both relationship and sexual partners [8]. In a study based in North Carolina, young Black MSM who sought sex partners via apps were more likely to report sexual minority stigma, racial discrimination, and perceived homophobia compared to app nonusers [9]. An Australian study found that sexual racism was associated with racist attitudes more broadly, challenging the “personal preferences” explanation of sexual racism [10]. Even on Jack’d, “colloquially referred to as the ‘hookup app for gay black men to get laid’” [11], racial preferences are sometimes expressed on profiles, with Asian men most preferred among White and Asian users and Black men most preferred among Black and Hispanic users in 1 study [12]. Despite substantial evidence that Black YMSM-YTW experience sexual racism in online dating, there is no evidence about whether this results in quantifiable differences in online sexual partnering by race and ethnicity.

Research consistently finds high levels of within–race and ethnicity sexual partnering among young MSM [7,13-17]. A recent study suggested that sexual exclusivity among Black sexual minority men may be partially protective against the psychological impacts of racial discrimination [18]. Within–race and ethnicity partnering impacts the structure of sexual networks and plays a part in driving continued disparities in HIV and other sexually transmitted infection (STI) prevalence between Black non-Hispanic YMSM-YTW and other races or ethnicities in the United States [19].

Despite this, we know relatively little of specific websites and mobile apps used by Black YMSM-YTW and how this use differs across races and ethnicities. Within a cohort of YMSM-YTW from Chicago, we aimed to characterize racial and ethnic differences (1) in the use of any website or mobile apps (hereafter referred to as “apps”) to find a sexual partner; (2) in the type of app used to find a sexual partner (eg, use of a “social network” as opposed to one predominantly used for dating, hookups, or escort services); (3) in which specific apps were used; and (4) among those who reported attempting to use an app to find a sexual partner and the successful meeting of a sexual partner through an app.

## Methods

### Participants and Procedures

Data presented in this study come from the PLOT ME (Plotting Layers of Transmission in Micro-Epidemics) supplemental study to the RADAR longitudinal cohort study. The RADAR cohort has followed a racially and ethnically diverse sample of YMSM-YTW living in the Chicago area every 6 months since 2015. Participants were recruited to RADAR through participation in prior cohorts of sexual and gender minority youth and adolescents (Project Q2 [20] and Crew 450 [21]), and through recruitment of new cohort members through venue-based, peer, or online recruitment or recruitment of serious partners of existing RADAR cohort members. Eligibility requirements for enrollment were as follows: aged 16‐29 years, assigned male sex at birth, English speaking, reported sex with a man in the prior year or identified as gay or bisexual, and able to attend study visits in Chicago. Further details of the RADAR study design have been previously published [22].

**Figure 1.**
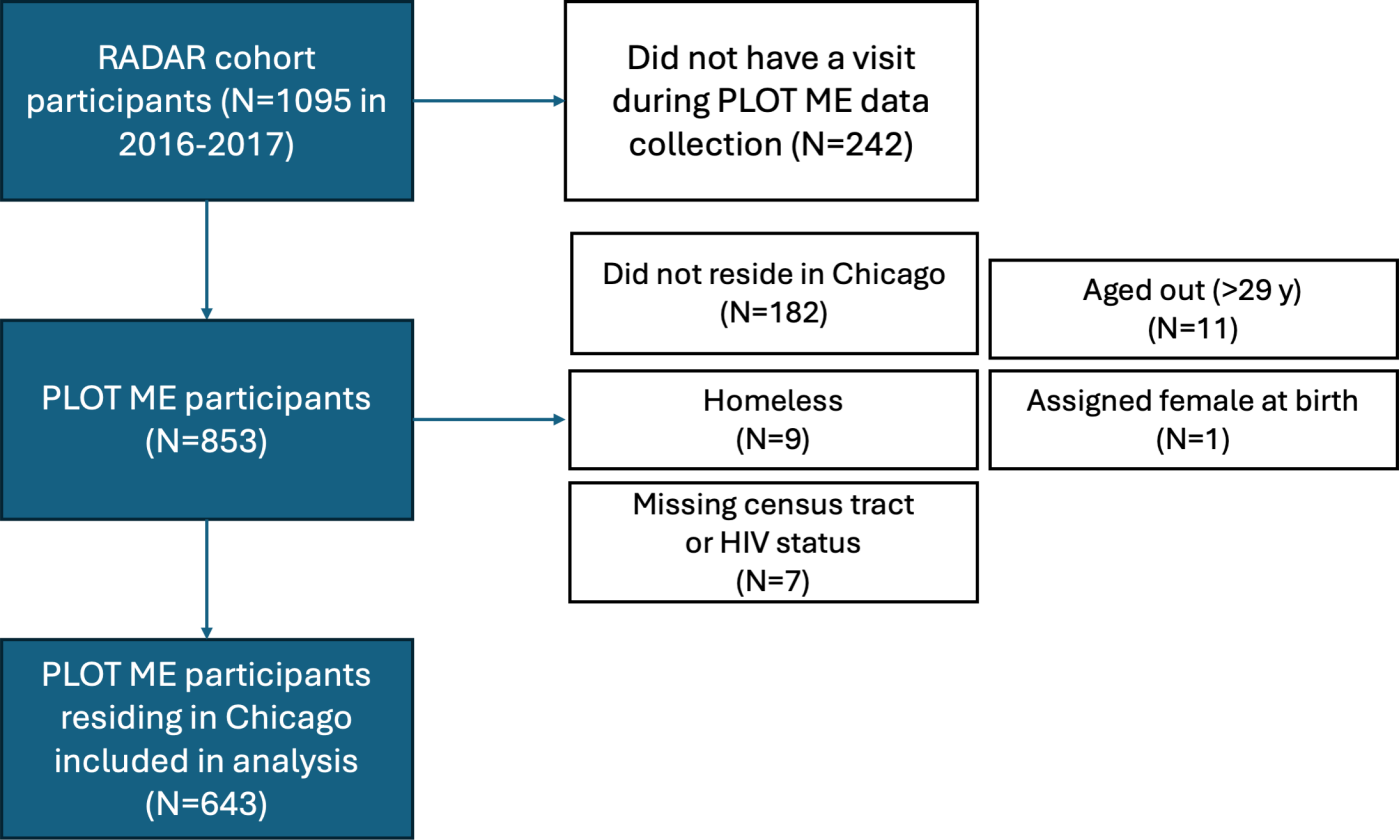
Flow diagram of RADAR cohort members, PLOT ME participants, and analytic sample for the presented cross-sectional study of dating or hookup app use among YMSM-YTW living in Chicago in 2016‐2017. PLOT ME: Plotting Layers of Transmission in Micro-Epidemics; YMSM-YTW: young men who have sex with men and young transgender women.

The PLOT ME supplemental study collected additional data on health care provider–, venue-, mobile app–, and neighborhood-level factors associated with HIV transmission. Data on venues attended to meet friends and sex partners, mobile apps and websites used to meet sex partners, and sexual health providers and services attended were collected using Network Canvas [23]. PLOT ME was administered to all participants in the RADAR cohort, with a study visit between September 6, 2016, and September 8, 2017. For participants who responded to the PLOT ME questions at multiple interviews, only data from the most recent interview were included. The analytic sample was restricted to people living in Chicago at the time of the interview who were not missing geographic data and HIV status, were aged 16‐29 years at the time of the interview, and were assigned male sex at birth. Study inclusion is detailed in Figure 1.

To guide our study questions, analysis, and interpretation of findings, our team relied upon a 5-member community advisory board (CAB) comprised of 2 Black-identified MSM, 2 Latinx-identified MSM, and 1 Black-identified transgender woman, all of whom work within or lead agencies concerned with sexual and gender minority health and HIV prevention in Chicago. Our CAB has proved instrumental in providing insight into the lived experiences of the communities being described.

### Measures

To assess app use, participants were asked “in the last 6 months, what websites or mobile apps did you use to meet people for sex, dating, or relationships?” Participants were allowed to nominate as many apps as they used and provided the names of apps through free response. Participants were also asked how often they had used each app (daily, weekly, monthly, or less than monthly). In analysis, apps were categorized as 1 of 3 types (“hookup or dating,” “social network,” or “classified and escort”) based on the marketing materials for the app and consultation with our CAB. Our first three outcomes were based on these data: (1) whether a participant reported any app use to meet a sexual partner in the prior 6 months, (2) among those who reported any use in the prior 6 months, the type of app used (“social network” vs all other types), and (3) among those who reported any use in the prior 6 months, which specific apps were used.

Participants were additionally asked to name or provide a nickname for all sexual partners in the prior 6 months via a network interview. Comprehensive details about this interview can be found elsewhere [24]. Using a sequence of name generators, social, drug, and sexual network members were captured. Follow-up questions focused on important network member attributes (eg, race) and important attributes of the connections between the participant and the network members (eg, estimated dates of first and last sex). For all sexual partners, participants were asked to indicate where this sexual partner was met, both broadly (categories were bar or club, web-based or mobile app, school, or somewhere else) and specifically (for partners met online, the specific app used was asked through free response). If a partner was met in multiple locations (eg, in person and online), we included that partner as having been met online. If a partner was listed as met in multiple apps, we categorized the partner as having been met in the first app listed. Our final outcome, defined among those who reported attempting to use specific apps to find a sexual partner, was 2 binary variables indicating whether the participant reported having successfully met a sexual partner in the prior 6 months using that specific app and overall.

Our primary independent variable of interest was race and ethnicity. At baseline, participants were asked their racial identity and whether they identified as Hispanic or Latinx. Participants were categorized first by ethnicity and then race into 4 racial and ethnic groups: White non-Hisparnic, Black non-Hispanic, Latinx, and other non-Hispanic. Non-Hispanic people who identified as 2 or more races are categorized as “other non-Hispanic.” Additional covariates of interest were sexual identity, gender identity, and age, which were all collected at baseline.

### Data Analysis

We present descriptive analyses of each outcome tabulated by the independent variables of interest (race and ethnicity, age, gender identity, and sexual identity). In descriptive analyses of which specific app was used and the successful meeting of a partner on a specific app by race and ethnicity, where cross-tabbed population sizes were sufficiently large (>5 respondents in each cell), we used a *χ*^2^ test, and in the case of small cell sizes, we used a Fisher exact test, to assess differences in outcome distribution by race and ethnicity. To assess differences in counts of apps reported by race and ethnicity, we used the Kruskal-Wallis test. We conducted simple and multiple logistic regression to assess the unadjusted and adjusted odds of 3 outcomes (any app use, type of app used, and the successful meeting of a partner on any app) by the independent variables of interest. In multiple logistic regression models, we included all independent variables of interest, regardless of unadjusted odds ratio (OR), based on a conceptual framework that age, gender identity, and sexual identity are likely confounders of the relationship between race and ethnicity and each app use–related outcome of interest. We additionally included frequency of app use (any daily use vs less than daily) in the model of meeting a partner to account for potential confounding. All analyses were conducted in R (R Foundation for Statistical Computing).

### Ethical Considerations

The RADAR cohort (STU00087614) and supplemental study (STU00206323) were approved by the Northwestern University Institutional Review Board. Informed consent or assent with a waiver of parental consent was obtained from all participants. While the RADAR data set includes identifiable information about participants for retention purposes, this is stored securely and separately from the deidentified data sets used for these analyses. As data were deidentified prior to receipt for this analysis and contained no information that linked the data to identifiable people, this was formally determined by the Northwestern Institutional Review Board as not human subjects research (STU00208999). Participants were compensated US $50 for each study visit.

## Results

### Sociodemographic Characteristics

At the most recent PLOT ME visit at which the respondent reported living in Chicago, a plurality of the 643 participants were Black non-Hispanic (274/643, 42.6%), and roughly one-quarter were White non-Hispanic (161/643, 25%) and one-quarter were Latinx (186/643, 28.9%; Table 1). A majority were aged 20‐24 (22/643, 53.8%; age: median 22.3, IQR 20.2‐24.9) years, and most identified as cisgender men (580/643, 90.2%) while roughly one-tenth identified as either transgender women (44/643, 6.8%) or another gender (19/643, 3%). Most identified as gay (452/643, 70.3%), and nearly one-third identified as either bisexual (110/643, 17.1%) or another sexual identity (81/643, 12.6%).

**Table 1.** Participant sociodemographic characteristics (n=643) in a cross-sectional study of app use for meeting sexual partners among YMSM-YTW living in Chicago in 2016‐2017.

Characteristics	Participants, n (%)	
**Race and ethnicity**		
Black non-Hispanic	274 (42.6)	
Latinx	186 (28.9)	
Other non-Hispanic	22 (3.4)	
White non-Hispanic	161 (25)	
**Age (years)**		
16‐19	141 (21.9)	
20‐24	346 (53.8)	
25‐29	156 (24.3)	
**Gender identity**		
Cisgender male	580 (90.2)	
Transgender female	44 (6.8)	
Other	19 (3)	
**Sexual identity**		
Gay	452 (70.3)	
Bisexual	110 (17.1)	
Other	81 (12.6)	

a YMSM-YTW: young men who have sex with men and young transgender women.

### Any App Use

Most participants (454/643, 70.6%) reported using 63 distinct apps to meet people for sex, dating, or relationships. Of the 63 apps, 43 were categorized as “hookup or dating” apps, 17 as “social network,” and 3 as “classified and escort.” App use was more common among White non-Hispanic (123/161, 76.4%), Latinx (135/186, 72.6%), and other races of non-Hispanic (18/22, 81.8%) participants than among Black non-Hispanic participants (178/274, 65%; Table 2). App use was greater among younger participants, decreasing from 82.3% (116/141) among 16‐ to 19-year-olds to 57.1% (89/156) among 25‐ to 29-year-olds. There were no significant differences in overall app use by gender identity or sexual identity. To assess whether these racial and ethnic differences in app use could be explained by different age distributions within the sample, we present adjusted ORs (aORs) of reporting any app use, and find that racial and ethnic differences are significant after adjustment. White non-Hispanic participants had 1.74 (95% CI 1.10‐2.76) times the odds of reporting app use compared to Black non-Hispanic participants after adjusting for age, gender identity, and sexual identity.

**Table 2.** Any app use, by demographic characteristics, in a cross-sectional study of mobile app use for meeting sexual partners among YMSM-YTW^a^ living in Chicago in 2016‐2017.

Demographics		App users, n/N (%)	Odds ratio (95% CI)	*P* value	Adjusted odds ratio^b^ (95% CI)	*P* value
**Race and ethnicity**				.04		.046
	Black non-Hispanic	178/274 (65)	Reference		Reference	
	Latinx	135/186 (72.6)	1.43 (0.95‐2.14)		1.32 (0.86‐2.02)	
	Other non-Hispanic	18/22 (81.8)	2.43 (0.8‐7.38)		2.67 (0.86‐8.24)	
	White non-Hispanic	123/161 (76.4)	1.75 (1.12‐2.71)		1.74 (1.1‐2.76)	
**Age (years)**				<.001		<.001
	16‐19	116/141 (82.3)	Reference		Reference	
	20‐24	249/346 (72)	0.55 (0.34‐0.9)		0.53 (0.32‐0.87)	
	25‐29	89/156 (57.1)	0.29 (0.17‐0.49)		0.29 (0.17‐0.5)	
**Gender identity**				.35		.43
	Cisgender male	406/580 (70)	Reference		Reference	
	Transgender female	32/44 (72.7)	1.14 (0.58‐2.27)		1.4 (0.64‐3.09)	
	Other	16/19 (84.2)	2.29 (0.66‐7.94)		2.01 (0.54‐7.49)	
**Sexual identity**				.33		.67
	Gay	312/452 (69)	Reference		Reference	
	Bisexual	80/110 (72.7)	1.2 (0.75‐1.9)		1.22 (0.76‐1.97)	
	Other	62/81 (76.5)	1.46 (0.84‐2.54)		1.16 (0.62‐2.19)	

a YMSM-YTW: young men who have sex with men and young transgender women.

b Adjusted model included race and ethnicity, age, gender identity, and sexual identity.

### Types of App Used—“Social Network” Apps Versus “Hookup or Dating” and “Classified” Apps

Among those who reported using apps to meet sexual or romantic partners in the prior 6 months, the type of app varied. Most respondents (88.5%, 402/454) reported using at least one “hookup or dating” app (eg, Grindr), 32.8% (149/454) reported using at least 1 “social network” app (eg, Facebook), and an additional 3.3% (15/454) reported using a “classified and escort” app (eg, Craigslist). There were racial and ethnic differences in type of app used, with much more common use of social network apps such as Facebook or Snapchat among Black non-Hispanic participants (83/178, 46.6%) than among White non-Hispanic (16/123, 13%), Latinx (46/135, 34.1%), or other racial groups (4/18, 22.2%). The difference between Black non-Hispanic and White non-Hispanic participants remained significant after adjusting for age, gender identity and sexual identity (Table 3), with White non-Hispanic participants being 20% as likely as Black participants to use “social network” apps to find partners versus “hookup or dating” and “classified and escort” apps (aOR 0.20, 95% CI 0.11‐0.37). Further, transgender women also had over 3 times the unadjusted odds of using “social network” apps to meet a partner compared to cisgender men (OR 3.25, 95% CI 1.56‐6.78), though these results did not remain statistically significant after adjusting for race and ethnicity, age, and sexual identity.

**Table 3.** Use of a “social network” app versus “hookup or dating” or “classified” app to meet sex partners, by demographic characteristics, in a cross-sectional study of mobile app use for meeting sexual partners among YMSM-YTW^a^ living in Chicago in 2016‐2017.

Demographics		Social network users, n/N (%)	Odds ratio (95% CI)	*P* value	Adjusted odds ratio^b^ (95% CI)	*P* value
**Race and ethnicity**				<.001		<.001
	Black non-Hispanic	83/178 (46.6)	Reference		Reference	
	Latinx	46/135 (34.1)	0.59 (0.37‐0.94)		0.65 (0.4‐1.05)	
	Other non-Hispanic	4/18 (22.2)	0.33 (0.1‐1.03)		0.34 (0.1‐1.09)	
	White non-Hispanic	16/123 (13)	0.17 (0.09‐0.31)		0.20 (0.11‐0.37)	
**Age (years)**				.01		.07
	16‐19	46/116 (39.7)	Reference		Reference	
	20‐24	67/249 (26.9)	0.56 (0.35‐0.89)		0.62 (0.38‐1.01)	
	25‐29	36/89 (40.4)	1.03 (0.59‐1.82)		1.03 (0.56‐1.87)	
**Gender identity**				.005		.20
	Cisgender male	126/406 (31)	Reference		Reference	
	Transgender female	19/32 (59.4)	3.25 (1.56‐6.78)		1.85 (0.79‐4.32)	
	Other	4/16 (25)	0.74 (0.23‐2.34)		0.61 (0.17‐2.22)	
**Sexual identity**				.32		.65
	Gay	96/312 (30.8)	Reference		Reference	
	Bisexual	28/80 (35)	1.21 (0.72‐2.03)		1.17 (0.68‐2.02)	
	Other	25/62 (40.3)	1.52 (0.87‐2.67)		1.34 (0.66‐2.69)	

a YMSM-YTW: young men who have sex with men and young transgender women.

b Adjusted model included race and ethnicity, age, gender identity, and sexual identity.

### Apps Used by Race and Ethnicity

Participants who reported using at least one app to try to meet partners reported using a median of 2 (IQR 1‐3, range 1‐18) apps in the past 6 months. There were no differences by race and ethnicity in the number of apps used (Kruskal-Wallis *P* value=.60). The most reported apps were Grindr (261/454, 57.5%), Tinder (156/454, 34.4%), and Jack’d (136/454, 30%). Characteristics of the 10 most frequently reported apps [25] are briefly described in Textbox 1. There were notable differences by race and ethnicity in app use (Table 4), with the 2 most common apps among White non-Hispanic participants (Grindr 93/123, 75.6% and Tinder 72/123, 58.5%) being substantially less common among Black non-Hispanic participants (Grindr 65/178, 36.5% and Tinder 25/178, 14%). The most common app reported among Black non-Hispanic participants was Jack’d (105/178, 59%). Only 4.9% (6/123) of White non-Hispanic participants reported using Jack’d, with a slightly higher proportion of Latinx (22/135, 16.3%) and other racial groups (3/18, 16.7%) reporting use of Jack’d.

Textbox 1. **Brief description of the 10 most frequently used apps for meeting sexual partners among young men who have sex with men and young transgender women living in Chicago.**

- Grindr is a geosocial networking mobile dating app that targets gay and bisexual men and transgender people, and presents potential matches based on geospatial distance from the user, and allows users to chat, “tap,” video call, and provide precise location to other users based on their personal profile. Profiles include a “looking for” section where users can indicate preferences. Must be aged 18+ years.
- Tinder is a geosocial networking mobile dating app that allows users to “swipe” to indicate interest, and requires both users to have indicated interest through swiping to be able to chat. Must be aged 18+ years.
- Jack’d is a geosocial networking mobile dating app that caters to gay and bisexual men, with a racially and ethnically diverse user base. Users view photos of other profiles sorted by geography, and are allowed to chat, “wave” at, share a private photo album with, or express “interest” in these other users, and users receive notifications when someone they are “interested” in reciprocates. Must be aged 18+ years.
- Facebook is a social networking site which allows people to interact with their “friends” using either public or direct messaging, along with a wide array of additional functionality. Does not incorporate geography. Must be aged 13+ years.
- Snapchat is a social network app that allows you to send messages, photos, or videos to “friends.” Messages disappear after 24 hours. Does not incorporate geography. Must be aged 13+ years.
- Instagram is a social network app that allows users to share images and videos, as well as direct instant messaging and video calling. Does not incorporate geography. Must be aged 13+ years.
- Scruff is a geosocial networking mobile dating app that targets gay and bisexual men and transgender and queer communities. Users see profiles sorted by geographic proximity, and can chat, send a “woof” or share private photo albums with other users. Must be aged 18+ years.
- OkCupid is an online dating site that matches users based on responses to a series of questions and allows users to enter their location but does not use a geosocial networking approach. Must be aged 18+ years.
- Plenty of Fish is an online dating site that allows users to set up a profile and view others’ profiles based on filtered characteristics. Users are able to “like” others profiles and if both like one another they are a “match.” Allows filtering on location but does not use a geosocial networking approach. Must be aged 18+ years.
- Adam4Adam is an online dating site predominantly for men seeking other men. Users browse others’ profiles based on up to 20 filters, send photos and messages to other users, and can favorite and “smile” at other users. Allows filtering on location but does not use a geosocial networking approach. Must be aged 18+ years.

**Table 4.** Apps used, by race and ethnicity and app type, in a cross-sectional study of mobile app use for meeting sexual partners among YMSM-YTW^a^ living in Chicago in 2016‐2017. Percent in parentheses reflects proportion of respondents who reported using each app.

App		Overall (N=454), n (%)	Black non-Hispanic (n=178), n (%)	Latinx (n=135), n (%)	Other non-Hispanic (n=18), n (%)	White non-Hispanic (n=123), n (%)	Fisher or*χ*^2^ *P* value
**Hookup or dating**							
	Grindr	261 (57.5)	65 (36.5)	91 (67.4)	12 (66.7)	93 (75.6)	<.001^b^
	Tinder	156 (34.4)	25 (14)	50 (37)	9 (50)	72 (58.5)	<.001^b^
	Jack’d	136 (30)	105 (59)	22 (16.3)	3 (16.7)	6 (4.9)	<.001
	Scruff	50 (11)	4 (2.2)	13 (9.6)	3 (16.7)	30 (24.4)	<.001
	OkCupid	30 (6.6)	7 (3.9)	9 (6.7)	0 (0)	14 (11.4)	.045
	Plenty of Fish	29 (6.4)	23 (12.9)	6 (4.4)	0 (0)	0 (0)	.001
	Adam4Adam	23 (5.1)	13 (7.3)	5 (3.7)	0 (0)	5 (4.1)	.30
	Badoo	17 (3.7)	14 (7.9)	2 (1.5)	1 (5.6)	0 (0)	.004
	GROWLr	15 (3.3)	5 (2.8)	4 (3)	1 (5.6)	5 (4.1)	.91
	Surge	12 (2.6)	6 (3.4)	1 (0.7)	1 (5.6)	4 (3.3)	.37
	Bumble	11 (2.4)	0 (0)	4 (3)	1 (5.6)	6 (4.9)	.03
	Hornet	11 (2.4)	2 (1.1)	5 (3.7)	0 (0)	4 (3.3)	.36
	Other hookup or dating app	73 (12.6)	26 (11.2)	24 (13.3)	4 (5.6)	19 (14.6)	.67
**Social network**							
	Facebook	106 (23.3)	64 (36)	31 (23)	3 (16.7)	8 (6.5)	<.001
	Snapchat	60 (13.2)	37 (20.8)	13 (9.6)	1 (5.6)	9 (7.3)	.002
	Instagram	51 (11.2)	28 (15.7)	15 (11.1)	1 (5.6)	7 (5.7)	.045
	Tumblr	11 (2.4)	6 (3.4)	4 (3)	1 (5.6)	0 (0)	.20
	Other social network	39 (7.3)	16 (8.4)	19 (10.4)	1 (5.6)	3 (2.4)	.08
**Classified and escort**							
	Craigslist	12 (2.6)	6 (3.4)	4 (3)	1 (5.6)	1 (0.8)	.44
	Other classified and escort	4 (0.9)	1 (0.6)	3 (2.2)	0 (0)	0 (0)	.31

a YMSM-YTW: young men who have sex with men and young transgender women.

b Superscript indicates *χ*2 test was used; all others are Fisher exact tests.

We further assessed specific app use by age, finding less striking differences by age than race and ethnicity (Table S1 in Multimedia Appendix 1). Older participants were less likely to report using Tinder (16/89, 18% among those aged 25‐29 y vs 47/116, 40.5% among those aged 16‐19 y and 93/249, 37.3%, among those aged 20‐24 y). In 9 respondents aged younger than 18 years (the age requirement for participation in most hookup or dating apps), 5 reported using a hookup or dating app.

### Successfully Meeting a Partner on an App

In this sample, most (569/643, 88.5%) respondents reported having any sexual partner in the previous 6 months, and over half (367/643, 57.1%) reported a new sexual partner in the time period. Of those with new partners, two-thirds reported having met any new partner online (241/367, 65.7%). Among those 241 people who had met any new partner online, there was a median of 1 (IQR 1‐3, range 1‐18) new partner met online.

Almost all respondents who met a new partner online in the prior 6 months also reported having used an app to meet people for sex, dating, or relationships (230/241, 95.4%). Of respondents who reported using an app to try to meet a partner, about half reported having successfully met a partner through an app (230/454, 50.7%). We next looked at the success of meeting a partner on an app, among those who reported having used a specific app to find a partner (Table S2 in Multimedia Appendix 1). The apps that people were most likely to have successfully met a partner on were Grindr (131/261, 50.2%), Scruff (21/50, 42%), and Craigslist (5/12, 41.7%). There were statistically significant differences between races or ethnicities in meeting a partner among all apps (explored more below). The only individual app where there were statistically significant differences between races or ethnicities without adjustment for other variables was Grindr, where a smaller proportion of Black non-Hispanic users successfully met partners than all other races or ethnicities (Black non-Hispanic 25/65, 38.5% vs White non-Hispanic 56/93, 60.2%, Latinx 43/91, 47.3%, and other non-Hispanic 7/12, 58.3%).

In multivariable regression analyses, we found a statistically significant difference by race and ethnicity in successfully meeting a new partner on an app among app users overall (Table 5). Across all apps, White non-Hispanic app users had over 2 times the odds of reporting having successfully met a sexual partner compared to Black non-Hispanic app users (aOR 2.46, 95% CI 1.50‐4.05), after adjusting for age, gender identity, sexual identity, and daily app use. We additionally found in the adjusted model that bisexual app users were substantially less likely to report successfully meeting a new partner compared to gay-identified app users (aOR 0.5, 95% CI 0.30‐0.84). We further assessed Grindr users specifically, again finding that White non-Hispanic users were more likely to report having successfully met a partner on Grindr than Black non-Hispanic Grindr users (aOR 2.57, 95% CI 1.30‐5.12), and bisexual Grindr users were less likely to report having successfully met a partner on Grindr than gay-identified Grindr users (aOR 0.33, 95% CI 0.14‐0.77; Table S3 in Multimedia Appendix 1).

**Table 5.** Comparison of meeting a partner in the past 6 months on an app, disaggregated by demographic groups, among those who reported using an app to find a partner in the prior 6 months, in a cross-sectional study of YMSM-YTW^a^ living in Chicago in 2016‐2017.

Demographics		Met partner, n/N (%)	Odds ratio (95% CI)	*P* value	Adjusted odds ratio^b^ (95% CI)	*P* value
**Race and ethnicity**				.002		.004
	Black non-Hispanic	73/178 (41)	Reference		Reference	
	Latinx	69/135 (51.1)	1.50 (0.96‐2.36)		1.46 (0.91‐2.34)	
	Other non-Hispanic	10/18 (55.6)	1.80 (0.68‐4.77)		1.83 (0.68‐4.97)	
	White non-Hispanic	78/123 (63.4)	2.49 (1.55‐4)		2.46 (1.50‐4.05)	
**Age (years)**				.22		.17
	16‐19	65/116 (56)	Reference		Reference	
	20‐24	126/249 (50.6)	0.8 (0.52‐1.25)		0.72 (0.45‐1.13)	
	25‐29	39/89 (43.8)	0.61 (0.35‐1.07)		0.59 (0.33‐1.06)	
**Gender identity**				.66		.93
	Cisgender male	207/406 (51)	Reference		Reference	
	Transgender female	14/32 (43.8)	0.75 (0.36‐1.54)		0.86 (0.37‐1.99)	
	Other	9/16 (56.2)	1.24 (0.45‐3.38)		0.90 (0.30‐2.72)	
**Sexual identity**				.02		.02
	Gay	166/312 (53.2)	Reference		Reference	
	Bisexual	29/80 (36.2)	0.50 (0.30‐0.83)		0.50 (0.30‐0.84)	
	Other	35/62 (56.5)	1.14 (0.66‐1.97)		1.26 (0.66‐2.4)	
**Any app used daily**				.38		.63
	Daily	124/254 (48.8)	Reference		Reference	
	Less than daily	106/200 (53)	1.18 (0.82‐1.71)		1.10 (0.74‐1.62)	

a YMSM-YTW: young men who have sex with men and young transgender women.

b Adjusted model included race and ethnicity, age, gender identity, sexual identity, and daily app use.

## Discussion

### Principal Findings

In this analysis, we found that Black non-Hispanic YMSM-YTW living in Chicago engaged with websites or mobile apps and found sexual partners on these apps systematically differently than other race and ethnicity groups, particularly White non-Hispanic YMSM-YTW. Black non-Hispanic YMSM-YTW were less likely to use an app to find sexual partners; if they did use an app, the apps used were often different from those used by White non-Hispanic YMSM-YTW and more likely to be a “social network” app (Table 6). Finally, if they did use an app, Black non-Hispanic YMSM-YTW were also less likely to successfully meet a sexual partner on an app. These results suggest racial and ethnic differences in the context of online sexual partnering for YMSM-YTW and have implications for driving the structure of sexual partnerships in ways which might contribute to racial and ethnic disparities in STIs.

**Table 6.** Summary of primary findings from multiple logistic regression analyses from a cross-sectional study of mobile app use for meeting sexual partners among YMSM-YTW^a^ living in Chicago in 2016‐2017.

	Any app use	Use of “social network” versus “dating” app	Successfully met a sex partner on app
Race and ethnicity	White YMSM-YTW had 1.74 times the odds of using apps versus Black YMSM-YTW	White YMSM-YTW had 0.20 times the odds of using a Social networking app to meet a sex partner versus Black YMSM-YTW	White YMSM-YTW had 2.46 times the odds of successfully meeting a sex partner versus Black YMSM-YTW
Age	Older YMSM-YTW less likely to use apps than older (0.53 for 20‐ to 24-year-olds and 0.29 for 25‐ to 29-year-olds, relative to 15‐ to 19-year-olds)	—^b^	—
Gender identity	—	—	—
Sexual identity	—	—	Bisexual-identified YMSM-YTW had 0.50 times the odds of successfully meeting a sex partner versus gay-identified YMSM-YTW

a YMSM-YTW: young men who have sex with men and young transgender women.

b Not applicable.

App use in general was less common among Black non-Hispanic YMSM-YTW. This is consistent with a recent systematic review which found that MSM app users (for social networking or sexual partner-seeking) were more likely to be younger and White than nonusers [26]. In part, experiences of discrimination in online dating may drive Black YMSM-YTW to avoid online dating [27]. Additionally, we found that increasing age is associated with a decreased likelihood of seeking a partner online. This is likely at least in part driven by the increased likelihood of having a stable partner in older populations.

We found that Black non-Hispanic YMSM-YTW were more likely to report using a social network app to find sexual partners, corroborating earlier qualitative work among Chicago young MSM [28]. In discussions with our CAB, this finding was unsurprising, as our members described that meeting sexual partners on “social network” apps felt safer particularly for Black people and transgender women, due to those sites being less anonymized than most dating apps, which allows for greater control of who you interact with (specifically “friends” instead of everyone on the app close to your geolocation). Our members also suggested that “social network” apps may give people more time to build connections than sites primarily organized around hookups, which could be potentially important for those experiencing internalized stigma around their sexual or gender identity. For example, unlike on a site such as Grindr, transgender women would not need to explain to their Facebook friends that they were transgender, as it would likely be already known. For others who may identify as straight, particularly in communities where same-sex sexuality is heavily stigmatized, having an app such as Grindr on your phone might not be feasible, whereas a social network site does not disclose your sexual identity.

Similar to previous work, we found that racial and ethnic minority YMSM-YTW report using apps to find sexual partners outside of Grindr [1,29,30]. In an online survey of diverse MSM aged 18‐64 years living in the United States and Puerto Rico conducted in 2014‐2015, Black MSM were more likely to frequently use Jack’d, Adam4Adam, and BGCLive than either White or Latino MSM [1]. Their study found a higher proportion of Latino MSM frequently used Grindr than either Black or White MSM, while we found a higher proportion of White non-Hispanic YMSM-YTW compared to Latinx YMSM-YTW. In a sample of Black young MSM in the southern United States, proportions of specific app use among users were higher than YMSM-YTW in our study, with a notably higher proportion of Adam4Adam use (42% compared to 7.3% of Black non-Hispanic YMSM-YTW in our study) [29]. In part, these differences in app use prevalence may be partly driven by data collection mechanisms, with our study using a free response name generator compared to the Duncan et al [29] study which used a closed form select all that apply approach. Overall, if they did use apps, Black MSM and transgender women frequently used different apps from White MSM and transgender women. These differences in app usage can lead to racial homophily in partnerships, which is one of the factors contributing to racial disparities in HIV prevalence [19].

Black non-Hispanic YMSM-YTW app users in our sample were significantly less likely to successfully form a sexual partnership online, relative to all other races and ethnicities. This is likely driven by sexual racism [2,4,7,14,27] among the predominantly White app user base. In prior work, across races and ethnicities, app using MSM expressed a preference for White and Hispanic men and a dis-preference for Black non-Hispanic and Asian men as both sexual and relationship partners [8]. Simultaneously, work finding that within-race sexual partnering protects from discrimination [18] suggests that Black participants who do not form partnerships despite using apps, including apps such as Grindr that are predominantly used by White people, may be choosing not to form partnerships on apps to protect themselves from discrimination.

### Limitations

There are several limitations to our analysis. First, the landscape of websites and mobile apps for hookups or dating has changed quickly, with some apps reported in our 2016‐2017 data collection being defunct, while new ones have become available and popular. Community members have advised us that Sniffies, WhatsApp, Telegram and Facebook Dating are all increasingly popular, while Craigslist Personals and Backpage no longer exist. Second, we dichotomized whether or not a partner was “successfully” found on an app. Given that people use different apps with different motivations and for different periods of time, this simplifies YMSM-YTW’s experience of apps. For instance, a study found that less than 30% of Grindr users listed meeting a sexual partner as the primary reason for their app use [31]. However, no studies we are aware of have previously characterized this per-app partnering rate and thus contributes to our understanding of how apps are used among YMSM-YTW. Third, in discussion with our CAB, community members noted that while the app types (eg, hookup or dating, social network) presented are the predominant way these apps are used, apps are multipurpose and may be used for multiple reasons (eg, Facebook could be used as an escort app, or Twitter can be used as a hookup app). Fourth, the RADAR cohort study from which our study participants are derived is not a random sample of the target population—YMSM-YTW living in Chicago. While we anticipate that the relative findings (eg, Black non-Hispanic relative to White non-Hispanic participants) likely hold in the target population, the nonrandom sampling approach may mean that descriptive estimates, such as the prevalence of specific apps used, may not be representative of our target population. Further, our findings are limited in generalizability to the Chicago area, and we hope to pursue comparisons to other geographic areas in future work. Finally, our sample size is not sufficiently large that we can make definitive conclusions about a lack of difference between racial and ethnic groups, particularly with respect to Latinx and other non-Hispanic groups.

### Conclusions

This study provides further evidence that the experience of Black non-Hispanic YMSM-YTW in finding sexual partners online is systematically different than other racial and ethnic groups. These findings provide insights for understanding the formation of partnerships among a key demographic group in the transmission of HIV and other STIs. A better understanding of how these apps are used and how they shape the sexual partnership of racially and ethnically diverse populations is important for understanding the mechanisms behind the disparities in HIV and other STIs.

## Supplementary material

10.2196/54215 Multimedia Appendix 1 Additional descriptive statistics and regression results.

## Abbreviations

aOR: adjusted odds ratio
CAB: community advisory board
MSM: men who have sex with men
OR: odds ratio
PLOT ME: Plotting Layers of Transmission in Micro-Epidemics
STI: sexually transmitted infection
YMSM-YTW: young men who have sex with men and young transgender women
