# Supplementary material for: Racial and Ethnic Differences in Mobile App Use for Meeting Sexual Partners Among Young Men Who Have Sex With Men and Young Transgender Women: Cross-Sectional Study
Source: JMIR Public Health Surveill. 2024 Sep 11;10:e54215. doi: 10.2196/54215 (PMC11409829; doi:10.2196/54215)
Supplement: Multimedia Appendix 1 [file publichealth-v10-e54215-s001.docx]

**Results tables:**

**Table S1.** Tabulation of apps by age. Percent reflects proportion of respondents reporting using an app or group of apps.

| App Type | App | Overall | 16-19  (n=116) | 20-24  (n=249) | 25-29  (n=89) | Chisq *P* |
| --- | --- | --- | --- | --- | --- | --- |
| Hookup/dating app | Grindr | 261 (57.5) | 63 (54.3) | 154 (61.8) | 44 (49.4) | .508 |
|  | Tinder | 156 (34.4) | 47 (40.5) | 93 (37.3) | 16 (18) | .675 |
|  | Jack’d | 136 (30) | 29 (25) | 74 (29.7) | 33 (37.1) | .051 |
|  | Scruff | 50 (11) | 6 (5.2) | 33 (13.3) | 11 (12.4) | .001 |
|  | OkCupid | 30 (6.6) | 4 (3.4) | 19 (7.6) | 7 (7.9) | .072 |
|  | PlentyofFish | 29 (6.4) | 4 (3.4) | 15 (6) | 10 (11.2) | .681 |
|  | Adam4Adam | 23 (5.1) | 2 (1.7) | 7 (2.8) | 14 (15.7) | .659 |
|  | Badoo | 17 (3.7) | 2 (1.7) | 10 (4) | 5 (5.6) | .092 |
|  | GROWLr | 15 (3.3) | 1 (0.9) | 7 (2.8) | 7 (7.9) | .172 |
|  | Surge | 12 (2.6) | 4 (3.4) | 5 (2) | 3 (3.4) | .125 |
|  | Bumble | 11 (2.4) | 3 (2.6) | 7 (2.8) | 1 (1.1) | .016 |
|  | Hornet | 11 (2.4) | 4 (3.4) | 4 (1.6) | 3 (3.4) | .288 |
|  | Other hookup/dating app | 73 (12.6) | 15 (10.3) | 36 (10.8) | 22 (20.2) | .065 |
| Social network | Facebook | 106 (23.3) | 32 (27.6) | 49 (19.7) | 25 (28.1) | .327 |
|  | Snapchat | 60 (13.2) | 22 (19) | 25 (10) | 13 (14.6) | .005 |
|  | Instagram | 51 (11.2) | 15 (12.9) | 28 (11.2) | 8 (9) | .058 |
|  | Tumblr | 11 (2.4) | 0 (0) | 4 (1.6) | 7 (7.9) | .257 |
|  | Other social network | 39 (7.3) | 12 (9.5) | 14 (5.6) | 13 (9) | .001 |
| Classified and escort | Craigslist | 12 (2.6) | 1 (0.9) | 4 (1.6) | 7 (7.9) | <.001 |
|  | Other classified and escort | 4 (0.9) | 0 (0) | 2 (0.8) | 2 (2.2) | .36 |

**Table S2.** Tabulation of meeting a new partner on an app by race or ethnicity. Percent reflects proportion of respondents who successfully met a partner out of those reporting using that app to meet a partner in the prior 6 months. NH = non-Hispanic.

| App | Overall | Black NH | White NH | Latinx | Other NH | Fisher’s/Chisq *P* | |
| --- | --- | --- | --- | --- | --- | --- | --- |
| All Apps | 230/454 (50.7) | 73/178 (41) | 78/123 (63.4) | 69/135 (51.1) | 10/18 (55.6) | .002^a^ | |
| Hookup/dating app | |  |  |  |  |  |  |
| Grindr | 131/261 (50.2) | 25/65 (38.5) | 56/93 (60.2) | 43/91 (47.3) | 7/12 (58.3) | .05^a^ | |
| Tinder | 39/156 (25) | 2/25 (8) | 23/72 (31.9) | 12/50 (24) | 2/9 (22.2) | .11 | |
| Jack’d | 30/136 (22.1) | 27/105 (25.7) | 0/6 (0) | 3/22 (13.6) | 0/3 (0) | .20 | |
| Scruff | 21/50 (42) | 0/4 (0) | 15/30 (50) | 4/13 (30.8) | 2/3 (66.7) | .15 | |
| OkCupid | 5/30 (16.7) | 1/7 (14.3) | 3/14 (21.4) | 1/9 (11.1) | 0/0 | .83 | |
| PlentyofFish | 3/29 (10.3) | 2/23 (8.7) | 0/0 | 1/6 (16.7) | 0/0 | 1 | |
| Adam4Adam | 2/23 (8.7) | 0/13 (0) | 0/5 (0) | 2/5 (40) | 0/0 | .08 | |
| Badoo | 3/17 (17.6) | 2/14 (14.3) | 0/0 | 0/2 (0) | 1/1 (100) | .22 | |
| GROWLr | 4/15 (26.7) | 1/5 (20) | 1/5 (20) | 2/4 (50) | 0/1 (0) | .68 | |
| Surge | 1/12 (8.3) | 0/6 (0) | 1/4 (25) | 0/1 (0) | 0/1 (0) | .50 | |
| Bumble | 0/11 (0) | 0/0 | 0/6 (0) | 0/4 (0) | 0/1 (0) | -^b^ | |
| Hornet | 0/11 (0) | 0/2 (0) | 0/4 (0) | 0/5 (0) | 0/0 | -^b^ | |
| Social network | |  |  |  |  |  | |
| Facebook | 13/106 (12.3) | 6/64 (9.4) | 0/8 (0) | 6/31 (19.4) | 1/3 (33.3) | .18 | |
| Snapchat | 3/60 (5) | 2/37 (5.4) | 1/9 (11.1) | 0/13 (0) | 0/1 (0) | .62 | |
| Instagram | 5/51 (9.8) | 2/28 (7.1) | 0/7 (0) | 3/15 (20) | 0/1 (0) | .40 | |
| Tumblr | 0/11 (0) | 0/6 (0) | 0/0 | 0/4 (0) | 0/1 (0) | -^b^ | |
| Classified and escort | |  |  |  |  |  | |
| Craigslist | 5/12 (41.7) | 2/6 (33.3) | 0/1 (0) | 2/4 (50) | 1/1 (100) | .85 | |

^a^Superscript indicates Chi-squared test was used, all others are Fisher’s exact tests. ^b^In apps with 0 successful matchings, comparison tests cannot be run.

**Table S3.** Comparison of meeting a partner on Grindr in the past 6 months disaggregated by demographic groups, among those who reported using Grindr in the prior 6 months.

| Demographics | App Users n/N | Percent | OR (95% CI) | *P* | aOR* (95% CI) | *P* |
| --- | --- | --- | --- | --- | --- | --- |
| Race / Ethnicity |  |  |  | .046 |  | .048 |
| Black non-Hispanic | 25/65 | 38.5 | REF |  | REF |  |
| White non-Hispanic | 56/93 | 60.2 | 2.42 (1.26-4.64) |  | 2.57 (1.3-5.12) |  |
| Latinx | 43/91 | 47.3 | 1.43 (0.75-2.74) |  | 1.46 (0.74-2.9) |  |
| Other non-Hispanic | 7/12 | 58.3 | 2.24 (0.64-7.83) |  | 1.91 (0.53-6.96) |  |
| Age |  |  |  | .344 |  | .323 |
| 16-19 | 36/63 | 57.1 | REF |  | REF |  |
| 20-24 | 76/154 | 49.4 | 0.73 (0.4-1.32) |  | 0.69 (0.37-1.3) |  |
| 25-29 | 19/44 | 43.2 | 0.57 (0.26-1.24) |  | 0.55 (0.24-1.25) |  |
| Gender identity |  |  |  | .37 |  | .079 |
| Cisgender Male | 125/246 | 50.8 | REF |  | REF |  |
| Transgender Female | 1/5 | 20 | 0.24 (0.03-2.19) |  | 0.1 (0.01-1.25) |  |
| Other | 5/10 | 50 | 0.97 (0.27-3.43) |  | 0.32 (0.06-1.62) |  |
| Sexual identity |  |  |  | .004 |  | .001 |
| Gay | 103/200 | 51.5 | REF |  | REF |  |
| Bisexual | 9/33 | 27.3 | 0.35 (0.16-0.8) |  | 0.33 (0.14-0.77) |  |
| Other | 19/28 | 67.9 | 1.99 (0.86-4.61) |  | 3.86 (1.2-12.37) |  |
| Frequency of use |  |  |  | .004 |  | .037 |
| Daily | 60/97 | 61.9 | REF |  | REF |  |
| Less than Daily | 71/164 | 43.3 | 0.47 (0.28-0.79) |  | 0.56 (0.32-0.97) |  |

**Adjusted model included race/ethnicity, age, gender identity, sexual identity, and daily app use*

**Table S4.** Tabulation of all apps reported.

| App | Count | Percent |
| --- | --- | --- |
| Grindr | 261 | 57.5 |
| Tinder | 156 | 34.4 |
| Jack’d | 136 | 30.0 |
| Facebook | 106 | 23.3 |
| Snapchat | 60 | 13.2 |
| Instagram | 51 | 11.2 |
| Scruff | 50 | 11.0 |
| OkCupid | 30 | 6.6 |
| PlentyofFish | 29 | 6.4 |
| Adam4Adam | 23 | 5.1 |
| Badoo | 17 | 3.7 |
| GROWLr | 15 | 3.3 |
| Craigslist | 12 | 2.6 |
| Surge | 12 | 2.6 |
| Bumble | 11 | 2.4 |
| Hornet | 11 | 2.4 |
| Tumblr | 11 | 2.4 |
| Kik | 10 | 2.2 |
| MeetMe | 9 | 2.0 |
| Recon | 9 | 2.0 |
| Tagged | 9 | 2.0 |
| Twitter | 8 | 1.8 |
| BarebackRT | 5 | 1.1 |
| BGCLive | 5 | 1.1 |
| Manhunt | 5 | 1.1 |
| Daddyhunt | 4 | 0.9 |
| Skout | 4 | 0.9 |
| UrbanCliq | 4 | 0.9 |
| Backpage | 3 | 0.7 |
| Bro | 3 | 0.7 |
| BoyAhoy | 2 | 0.4 |
| SeekingArrangement | 2 | 0.4 |
| Yellow | 2 | 0.4 |
| YouTube | 2 | 0.4 |
| Zoosk | 2 | 0.4 |
| Autistic Dating | 1 | 0.2 |
| Clover | 1 | 0.2 |
| Distinc.tt | 1 | 0.2 |
| eHarmony | 1 | 0.2 |
| Eros | 1 | 0.2 |
| FarmersOnly | 1 | 0.2 |
| Feeld | 1 | 0.2 |
| FetLife | 1 | 0.2 |
| Flirt | 1 | 0.2 |
| FWB Dating Only | 1 | 0.2 |
| GDaddy | 1 | 0.2 |
| hi5 | 1 | 0.2 |
| LinkedIn | 1 | 0.2 |
| Live.me | 1 | 0.2 |
| Match.com | 1 | 0.2 |
| Meetup | 1 | 0.2 |
| My Transgender Date | 1 | 0.2 |
| Nasty Kink Pigs | 1 | 0.2 |
| Only Lads | 1 | 0.2 |
| PartyLine | 1 | 0.2 |
| PHHHOTO | 1 | 0.2 |
| Pinterest | 1 | 0.2 |
| ReelFish | 1 | 0.2 |
| RentMen.com | 1 | 0.2 |
| Squirt | 1 | 0.2 |
| VIGR | 1 | 0.2 |
| Wapo | 1 | 0.2 |
| YouNow | 1 | 0.2 |
